# Microwave Radar Sensing for Non-Invasive Intra-Abdominal Pressure Monitoring: A Simulation-Based Analysis with Phantom Testing

**DOI:** 10.3390/s26175452

**Published:** 2026-08-28

**Authors:** Salar Tayebi, Ashkan Zarghami, Cheng Chen, Wojciech Dabrowski, Manu L. N. G. Malbrain, Johan Stiens

**Affiliations:** 1 Department of Electronics and Informatics, Vrije Universiteit Brussel, 1050 Brussel, Belgium; ashkan.zarghami@vub.be (A.Z.); cheng.chen@vub.be (C.C.); johan.stiens@vub.be (J.S.); 2 International Fluid Academy, 3360 Lovenjoel, Belgium; manu.malbrain@umlub.pl; 3 School of Information Science and Technology, Northwest University, Xi’an 710127, China; 4 First Department of Anaesthesiology and Intensive Therapy, Medical University of Lublin, 20-090 Lublin, Poland; wojciech.dabrowski@umlub.edu.pl; 5 Medical Data Management, Medaman, 2440 Geel, Belgium

**Keywords:** transient microwave reflectometry, intra-abdominal pressure, non-invasive measurement, numerical simulation, phantom testing

## Abstract

Background: Intra-abdominal pressure (IAP) has recently been recognized as a new vital sign in critically ill patients. Microwave reflectometry has been proposed as a potential approach for non-invasive IAP measurement. However, systematic investigation on how individual anatomical and geometric factors influence changes in the microwave reflection response of the abdominal compartment is limited. Complementary information regarding illumination frequency and specific absorption rate (SAR) also warrants consideration. Objective: This study aimed to advance the current knowledge on using microwave radar-based sensors in IAP monitoring by studying the most influencing factors. The penetration depth and spot size versus radiation frequency is studied as well. Information on energy deposition due to radio-frequency exposure is investigated too. Methods: Numerical simulations were performed using abdominal models adjusted to represent different IAP levels. Reflection signal features were analyzed in relation to IAP-induced changes, and SAR was calculated using human models. Subsequently, a radar sensor prototype was tested on a benchtop abdominal phantom. Lin’s concordance correlation analysis was used to evaluate absolute agreement between radar-estimated IAP and reference IAP. Additional statistical analyses assessed bias, precision, concordance, and risk levels. Results: Sagittal abdominal diameter was the dominant factor affecting the microwave reflection response. Reflection amplitude showed a periodic trend consistent with abdominal displacement corresponding to multiples of half-wavelength values of the applied electromagnetic waves. Numerical SAR simulations showed increasing SAR with frequency while remaining below the applicable exposure limits under the investigated conditions. The radar sensor showed a bias of 0.43 mmHg and a precision of 2.55 mmHg. Concordance analysis among the paired changes remaining after application of the predefined exclusion criteria showed agreement in the direction of IAP change. Conclusion: The present study should be considered a preliminary proof of concept. Clinically, the technology is currently more suitable for early warning and trend monitoring than for precise absolute IAP measurement, and it does not yet replace standard intravesical measurements. Its ability to support clinical decision-making, including guiding fluid therapy, requires prospective validation in patients.

## 1. Introduction

Intra-abdominal pressure (IAP), defined as the steady-state pressure within the abdominal compartment measured at end-expiration, is an important determinant of perfusion to abdominal and distal organs, as abdominal perfusion pressure is a function of mean arterial pressure and IAP [1]. Intra-abdominal hypertension (IAH) is defined as a sustained increased IAP equal to or above 12 mmHg [1]. Previous studies have shown a high prevalence of IAH in specific high-risk groups, including patients undergoing cardiac surgery (31.8%), those with major burns (53%), trauma (36%), liver cirrhosis (82.1%), severe acute pancreatitis (72.2%), general surgery (67.3%), and sepsis (43.5%) [2,3,4]. IAH can reduce perfusion of abdominal organs, contributing to kidney dysfunction and potentially affecting other organ systems [3,5,6,7,8]. If not identified early, it may progress to abdominal compartment syndrome (ACS), which is characterized by sustained IAH above 20 mmHg associated with new onset organ failure. The presence of ACS results in increased morbidity and mortality [1]. Therefore, early detection of IAH, followed by appropriate patient management to prevent progression to ACS, has the potential to improve treatment efficiency and reduce ICU length of stay. In addition, continuous IAP monitoring may provide complementary information on IAP trends during fluid resuscitation, which could potentially support the assessment of fluid responsiveness and tolerance [2]. These considerations emphasize the need for continuous IAP monitoring to facilitate timely recognition of IAH and appropriate clinical management.

Current reference measurements of IAP rely on the intra-bladder technique [1]. This gold-standard method, recommended by the Abdominal Compartment Society (WSACS, formerly known as the World Society of Abdominal Compartment Syndrome, www.wsacs.org and https://wsacs.mn.co (accessed on 25 August 2026)), recommends the use of a Foley catheter inserted into the urinary bladder to estimate the pressure within the abdominal cavity. Via a regular Foley catheter, IAP can be assessed either by observing the height of the urine column in the drainage tubing (the Foleymanometer technique [9]) or by transmitting the pressure waveform to an external pressure transducer positioned at the level where the mid-axillary line crosses the iliac crest with the patient in the supine position [1]. However, this method can be cumbersome for ICU staff and is invasive, carrying risks such as catheter-associated urinary tract infection (CAUTI) and trauma. It is also unsuitable for patients with bladder or pelvic tumors or trauma [10]. An additional consideration is bladder filling volume, as previous studies have shown that IAP readings are influenced by the amount of urine (or sterile saline) within the bladder [1]. These limitations have contributed to growing interest in developing continuous, non-invasive alternatives to the current gold-standard approach for IAP measurement.

Several indirect IAP measurement methods have been proposed in recent years that could potentially serve as alternatives [11,12,13,14,15,16]. The use of microwave reflectometry has been one of the promising approaches explored in this field. A group of researchers has investigated the use of frequency-based microwave reflectometry in porcine and human subjects and reported a strong correlation between IAP and the wideband reflection response of the abdominal wall (S11) in the 3.90–4.45 GHz range [17,18]. In another study, researchers explored the use of frequency-modulated continuous-wave (FMCW) radar at 60–64 GHz in porcine models and concluded that, with appropriate learning models, the reflection response of the abdominal wall can serve as a surrogate for IAP estimation [19]. Although previous studies have demonstrated a good correlation between IAP and changes in the abdominal reflection response, no study has systematically investigated how individual anatomical and geometric factors contribute to these changes during IAH. Complementary information regarding the appropriate illumination frequency for such an application is also needed. In addition, safety-related aspects such as specific absorption rate (SAR) warrant further consideration.

Accordingly, the primary aim of this research is to conduct a series of numerical simulations using Microwave Studio Suite (Dassault Systèmes, Ile-De-France, France) to examine abdominal compartment curvature (shape), abdominal wall thickness, and abdominal wall displacement (sagittal abdominal diameter) as the three key morphological and geometric changes during IAH, and to determine which of these factors most strongly influences the abdominal reflection signal. By performing these simulations at four different frequencies, the penetration depth and spot size (lateral resolution) are qualitatively studied as functions of frequency. Different reflection signal features have also been investigated in relation to IAP changes. In addition, using the software’s built-in human models, the SAR is calculated for subjects with different anthropometric characteristics to provide insight into safety considerations. Following these in silico investigations, the second part of the study will focus on the practical development of a transient-time-domain microwave-radar sensor and its evaluation on a bench-top abdominal phantom capable of simulating various IAH scenarios.

The novelty of this study lies in systematically decomposing the IAP-induced microwave reflection response into the individual contributions of abdominal wall displacement, thickness, and curvature, and in linking these simulation-derived mechanisms to specific radar signal features. The subsequent phantom experiments further assess whether these observations are reproduced in a physical sensing setup, providing a framework for the development of microwave-based IAP monitoring systems. Complementary numerical analyses of illumination frequency, tissue penetration, spot size, and SAR were additionally performed to provide practical guidance for sensor design and future clinical implementation.

## 2. Materials and Methods

### 2.1. Finite Element Simulations

#### 2.1.1. Abdominal Wall Model at Baseline IAP

**Figure 1:**
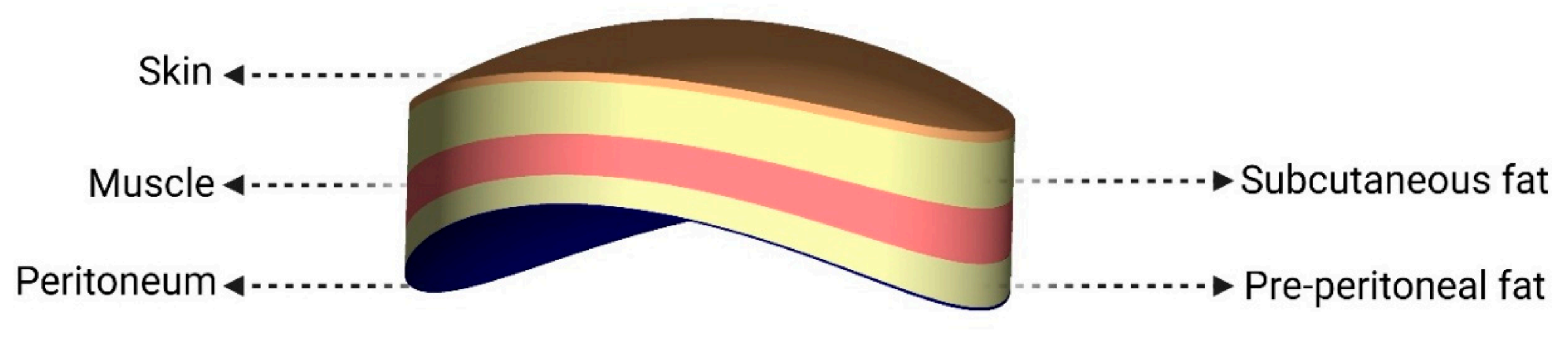
Computer-aided design (CAD) model of the abdominal wall at baseline IAP. As shown, it consists of five layers: skin, fat, muscle, preperitoneal fat, and peritoneum.

The abdominal wall at baseline IAP was modeled as a multilayer dielectric slab composed of skin, subcutaneous fat, muscle (rectus abdominis), preperitoneal fat, and peritoneum. Anatomical and mechanical properties for each layer were extracted from the literature [20,21,22,23,24,25,26,27,28] (Table 1) and used to construct a computer-aided design (CAD) model of the abdominal wall (Figure 1) in SolidWorks 2019 (Dassault Systèmes, Ile-de-France, France).

Next, the electromagnetic properties of the abdominal wall, including dielectric permittivity and electrical conductivity for each layer at 5, 10, 15, and 20 GHz were obtained from the literature [29,30] (Table 2) and assigned to each layer.

The final model was considered as the abdominal wall at baseline IAP. The model was further modified (as explained in the following subsection) to represent the abdominal wall at other IAP levels and to reflect different IAH grades.

#### 2.1.2. Abdominal Morphology Versus IAP

In order to mimic the morphological influence of IAP on the abdominal compartment, we needed to first understand how abdominal morphology changes during IAP elevation. IAP fluctuations affect the abdominal reflection response by three primary mechanisms, including displacing the abdominal wall by changing the sagittal abdominal diameter, changing the abdominal wall thickness, and altering the curvature (shape) of the abdominal wall.

**Figure 2:**
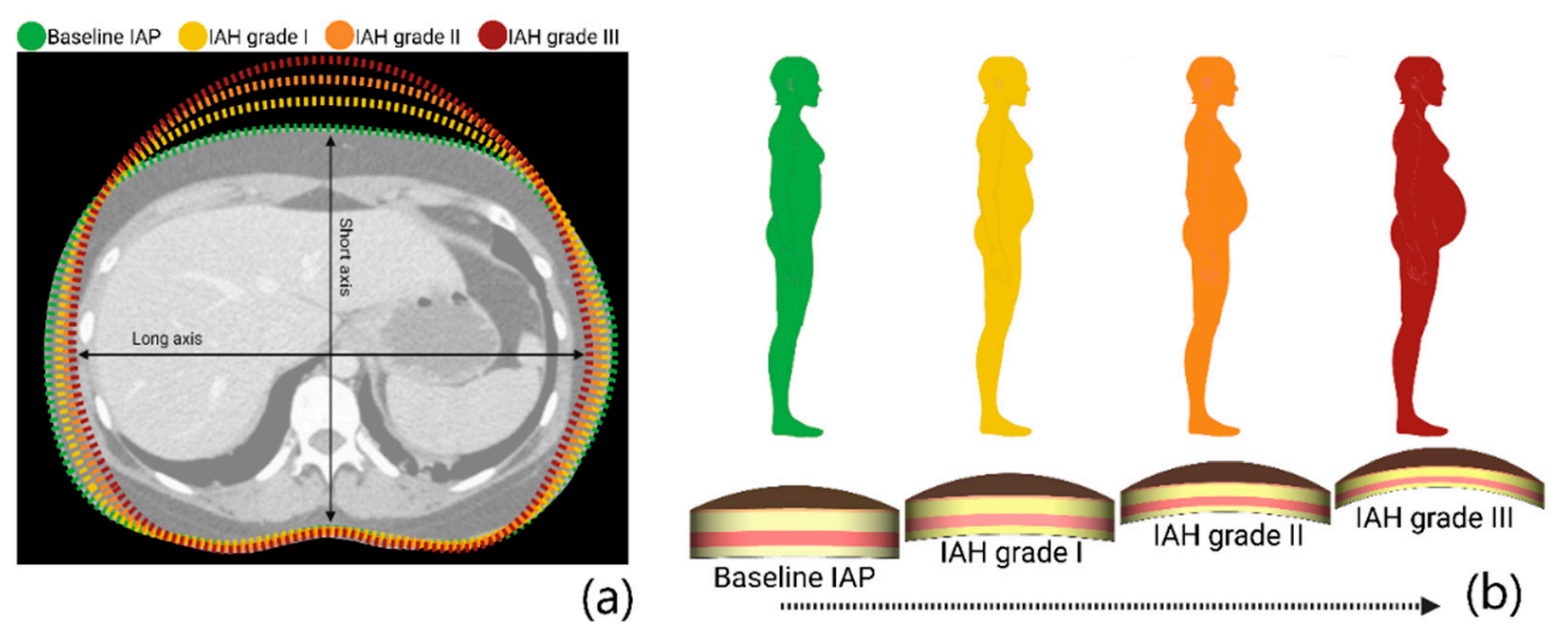
The evolution of the abdominal wall during IAP elevation. (**a**) Illustration of the abdominal short axis (sagittal abdominal diameter) and long axis (coronal abdominal diameter). (**b**) Different models of the abdominal wall representing IAH grades I, II, and III.

In general, the abdominal compartment can be assumed as an elliptical chamber at baseline IAP. However, as IAP increases, the elliptical shape becomes more spherical, altering the abdominal wall’s shape, position, and thickness. Previous studies on 96 ICU patients show that the long axis of the abdominal compartment (coronal abdominal diameter) decreases 1.33 ± 0.57 cm with every 5 mmHg of IAP elevation, on average [31]. In contrast, the short axis of the abdominal compartment (sagittal abdominal diameter) increases by a value of 3.33 ± 0.60 cm for every 5 mmHg of IAP increase (Figure 2). Regarding thickness changes, available ultrasound data from a study on 14 critically ill patients show that IAP elevation decreases abdominal wall thickness by a value of −0.12 ± 0.10 cm/mmHg [18].

Using values reported in the literature, the expected morphological changes associated with increasing IAP were applied to the baseline model to represent the abdominal compartment and wall at 10 mmHg (baseline), 15 mmHg (IAH grade I), 20 mmHg (IAH grade II), and 25 mmHg (IAH grade III). These models were then used to evaluate the reflection response of the abdominal compartment under simultaneous variation of all parameters, enabling a global (multivariate) sensitivity analysis.

In addition to the globally modified models, in which all three influencing factors were changed simultaneously, the baseline model was also adjusted by varying only one influencing factor at a time. Therefore, for each IAH level, three additional models were generated to represent conditions in which only a single parameter, including thickness, curvature, or distance, was altered. These simulations provided a local (univariate) sensitivity analysis. This approach enabled the identification of the parameter with the greatest influence on the reflected radar signal.

#### 2.1.3. Simulation Setup

A finite element simulation is a numerical modelling technique in which a sample under study is divided into a large number of small, simple subdomains (finite elements). The governing physical equations are solved locally within each element and then assembled to approximate the behaviour of the entire system. In this regard, electromagnetic simulations were done using Microwave Studio Suite (Dassault Systèmes, Ile-De-France, France). For all simulations, the microwave illumination towards the abdominal wall was simulated using a circular waveguide port with an aperture diameter of 8 cm, representing a circular horn antenna. The waveguide port was placed within the Fresnel’s region with respect to the front side of the abdominal wall model. A sine step excitation signal with a rise-time of 1 ns from zero illumination power to steady-state, and the total radiation time of 7 ns was fed into the waveguide port. Having this total radiation duration enabled us to check the transient and also the steady-state reflection response of the abdominal wall. An open boundary condition was applied in the X and Y (in-plane) directions, and an open-add-space boundary condition was applied in the Z (out-of-plane) direction. Since the abdominal wall model was symmetric, a symmetric magnetic field boundary condition was added to the Y-Z plane. In a similar way, a symmetric electric field boundary condition was assigned to the X-Z plane to lower the computation time. Lastly, the hexahedral mesh with a density of 20 cells per wavelength was assigned to run each simulation. For each simulation, the reflection response of the abdominal wall model, as well as the visualized electric field and power distribution through the layers, was recorded.

**Figure 3:**
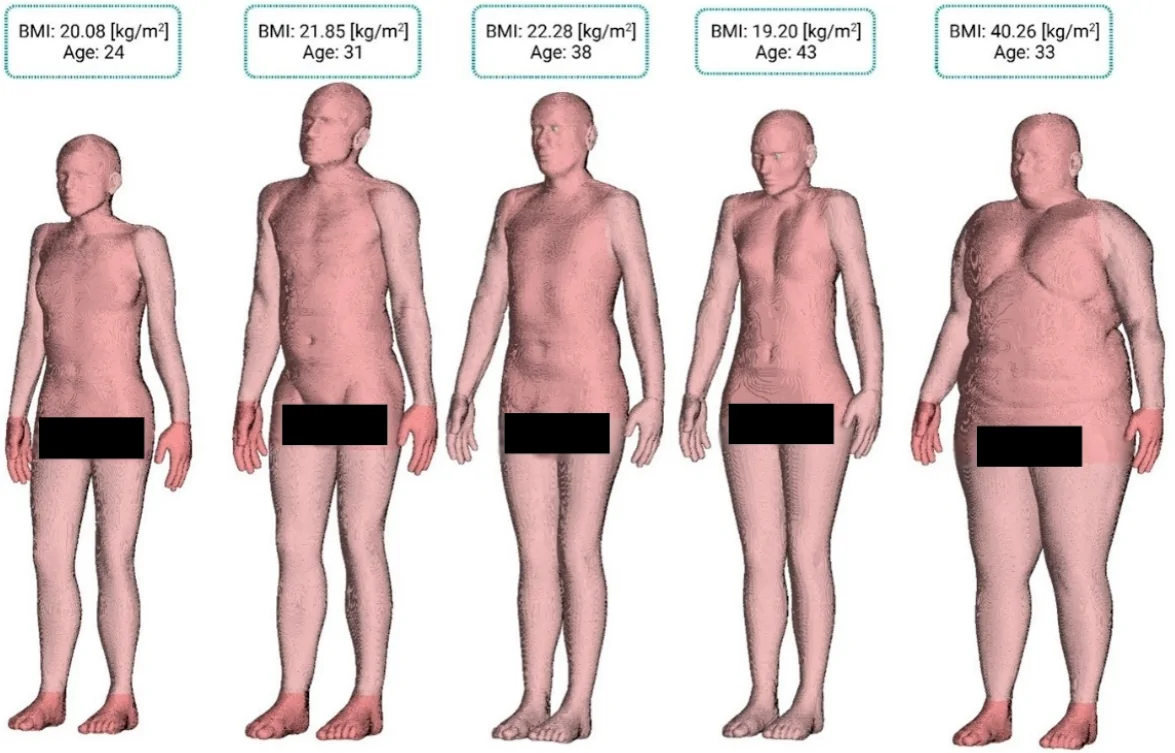
Virtual human models used for specific absorption rate (SAR) simulations.

For the SAR analysis, five virtual subjects from the software’s bio-model library (Figure 3), representing different ages and body mass index (BMI) values of 19.2, 20.1, 21.9, 22.3, and 40.2 kg/m^2^, were used to evaluate SAR in the abdominal wall during microwave exposure. For these simulations, the electromagnetic ports were positioned at the antenna-to-umbilical distance corresponding to an IAP of 25 mmHg, representing the maximum abdominal distension considered in this study. These bio-models are derived from pseudonymized CT images of real human bodies, and thus reflect realistic anatomical variability. Using the IEEE/IEC 62704-1 averaging method, 10 g SAR was calculated for the abdominal region of each virtual subject.

#### 2.1.4. Data Processing

For each IAP level, two types of simulation models were available: (1) multivariate models, in which all three morphological parameters were varied simultaneously; and (2) univariate models, in which only one parameter was varied while the remaining two were kept at their baseline values.

The time-domain reflection waveform S(t) was extracted for all models. To determine which morphological parameter contributes most strongly to the overall reflection signal, the similarity between each univariate response and the corresponding multivariate response was quantified using the normalized root-mean-square error (nRMSE):

(1) $$\mathrm{nRMSE}=\frac{\sqrt{\frac{1}{N}\sum_{i=1}^{N}{\left(S_{\mathrm{multi}}(t_{i})-S_{\mathrm{uni}}(t_{i})\right)}^{2}}}{max(S_{\mathrm{multi}})-min(S_{\mathrm{multi}})}$$

where $S_{\mathrm{multi}}\;(t)$ is the reflection signal from the multivariate model, $S_{\mathrm{uni}}\;(t)$ is the signal from a univariate model (displacement-only, thickness-only, or curvature-only), and *N* is the number of data points. In other words, each datapoint of $S_{\mathrm{multi}}\;(t)$ was compared with $S_{\mathrm{uni}}\;(t)$ to obtain the difference between them. A lower nRMSE indicates a closer match between the univariate and multivariate signals, signifying that the corresponding morphological parameter exerts a greater influence on the overall reflection response at that IAP level.

**Figure 4:**
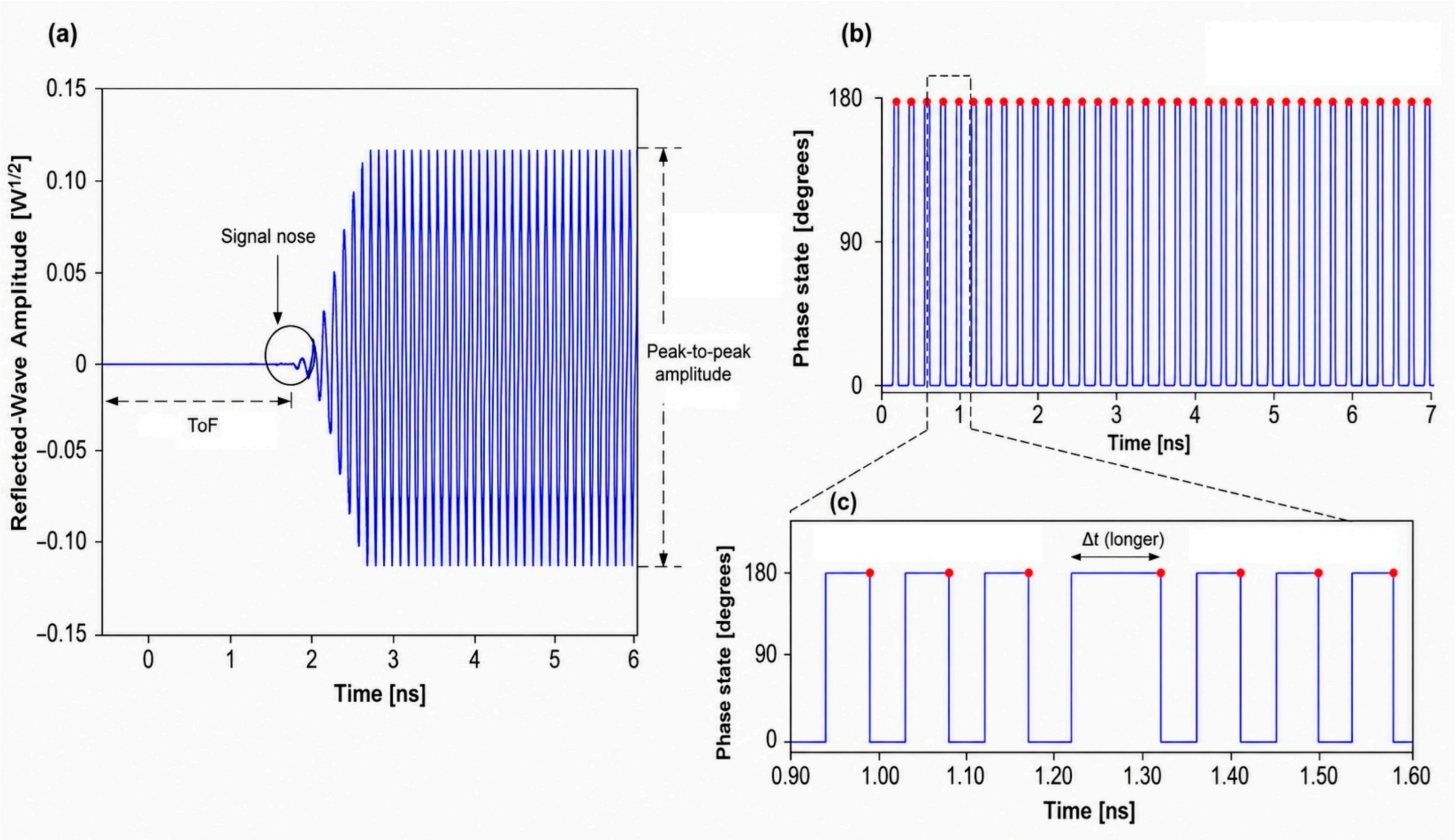
Analyzed reflection response features versus abdominal morphological changes. (**a**) The signal nose was monitored to extract the time-of-flight (ToF) feature, while the maximum peak-to-peak reflection amplitude was also assessed. (**b**) The phase-state representation of the reflected signal was evaluated at the receiver position. (**c**) A magnified view illustrates the variation in phase-state duration (Δt) around the signal nose.

In addition to the sensitivity analyses, the reflection signals were examined to assess how specific signal features change with increasing IAP. The signal nose, defined as the point at which the microwaves first encounter the anterior surface of the abdominal wall and reflect back, was evaluated as it provides time-of-flight (ToF) information. Along with ToF, the reflected-wave amplitude was analyzed using the maximum peak-to-peak amplitude. In addition, the phase state of the reflected waveform was calculated yielding 0° and 180° states corresponding to positive and negative signal values, respectively. A change in the phase-state duration therefore reflects a local phase shift/jump in the transient reflected waveform (Figure 4).

SAR values obtained from each virtual subject were averaged and reported as mean ± standard deviation (SD).

Statistical analyses were performed with MATLAB R2023a (The MathWorks Inc., Natick, MA, USA).

### 2.2. Bench-Top Phantom Testing

#### 2.2.1. Radar Sensor Development

**Figure 5:**
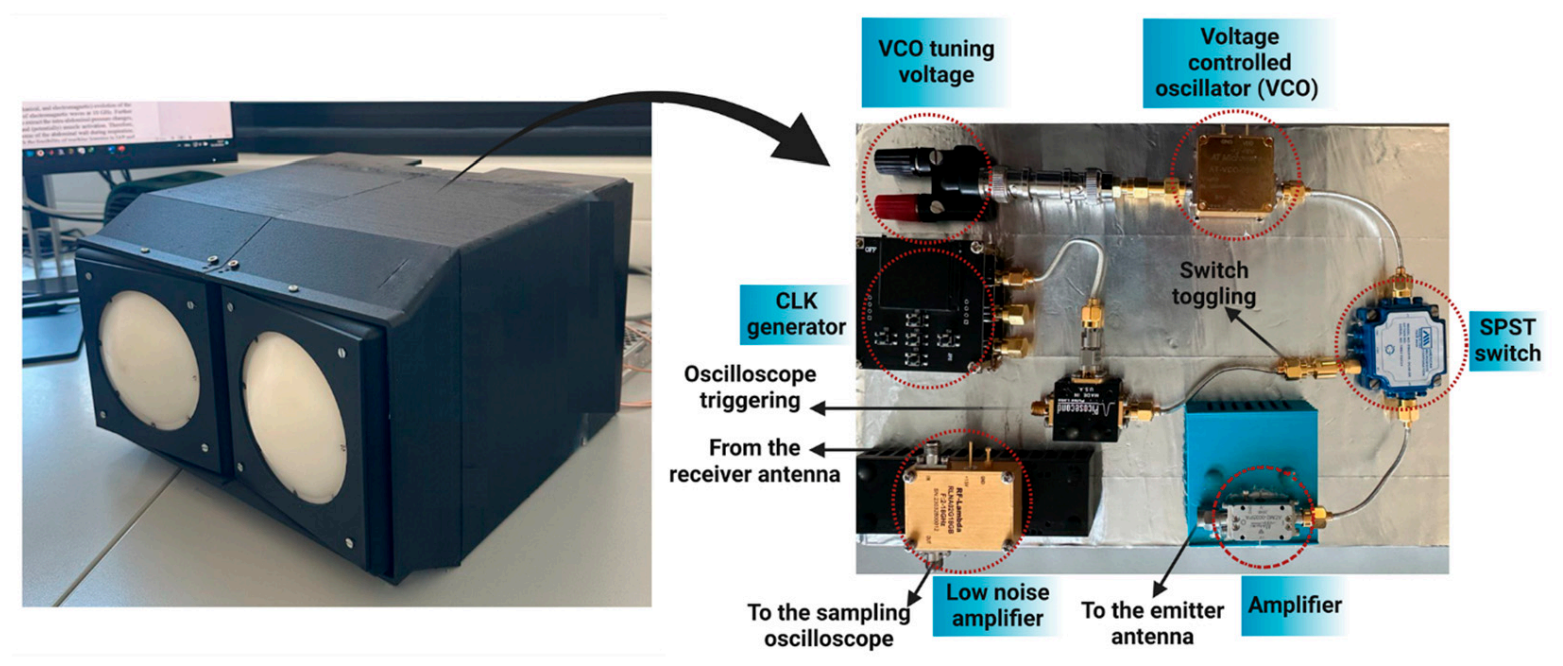
Microwave radar-based sensor on the basis of transient radar methodology. The carrier frequency was tuned by the voltage-controlled oscillator (VCO). Using a single-pole-single-throw (SPST) switch, the VCO’s output was modulated by the modulation signal from the system clock (CLK). Two amplifiers (one before emission and one after reception) were used to further increase the signal-to-noise ratio.

Following the first part of the study, we proceeded to develop a transient microwave radar sensor. The sensor architecture is shown in Figure 5 [32]. The system begins with a voltage-controlled oscillator (VCO; Shanghai AT Microwave Limited, Shanghai, China), which generates electromagnetic waves whose frequency can be finely adjusted via the input voltage. In this setup, the VCO input voltage was set to produce a carrier frequency of 10 GHz. Downstream of the VCO, a single-pole-single-throw (SPST) switch (American Microwave Corporation, Frederick, MD, USA) modulates the signal and can be toggled ON or OFF by an external pulse. For this purpose, an Si5351A clock generator (Skyworks Solutions, Inc., Irvine, CA, USA) operating at 8 MHz was used to control the switch. This stage provides the modulation necessary to generate excitation signals consistent with those used in the in silico studies. Following the SPST switch, an amplifier (Marki Microwave, Morgan Hill, CA, USA) increases the output power and improves the signal-to-noise ratio. Using this setup, the peak transmitter output power delivered to the transmitting antenna was 0.3 W. The amplified signal is then delivered to a transmitter antenna (Eravant, Torrance, CA, USA) and radiated toward the umbilical region of the abdominal wall. The reflected waves are captured by a receiver antenna and subsequently passed through a low-noise amplifier (Shanghai AT Microwave Limited, Shanghai, China) before being fed into a sampling oscilloscope (Pico Technology, Cambridgeshire, UK) for acquisition and analysis.

#### 2.2.2. Abdominal Phantom and Study Design

The abdominal phantom (Ventra) used in this study has been extensively described in previous studies [33]. To further adapt the phantom for electromagnetic sensing studies, the phantom’s skin and fat were replaced with an artificial skin and fat layer (SPEAG, Zurich, Switzerland) that mimics the electromagnetic properties of real human skin and fat. This multilayer abdominal wall features artificial skin (4 mm thick), subcutaneous fat (13 mm thick), muscle with fascia (10 mm thick), and peritoneum (less than 1 mm thick). The developed sensor was positioned in front of the Ventra’s abdominal wall at the Fresnel’s region. The IAP was changed between 5 and 35 mmHg with 10 repetitions, and the Ventra’s reflection response was continuously recorded at 5 Hz (every 0.2 s). The Ventra’s pressure sensor readings were used as reference IAP.

#### 2.2.3. Signal Processing

**Figure 6:**
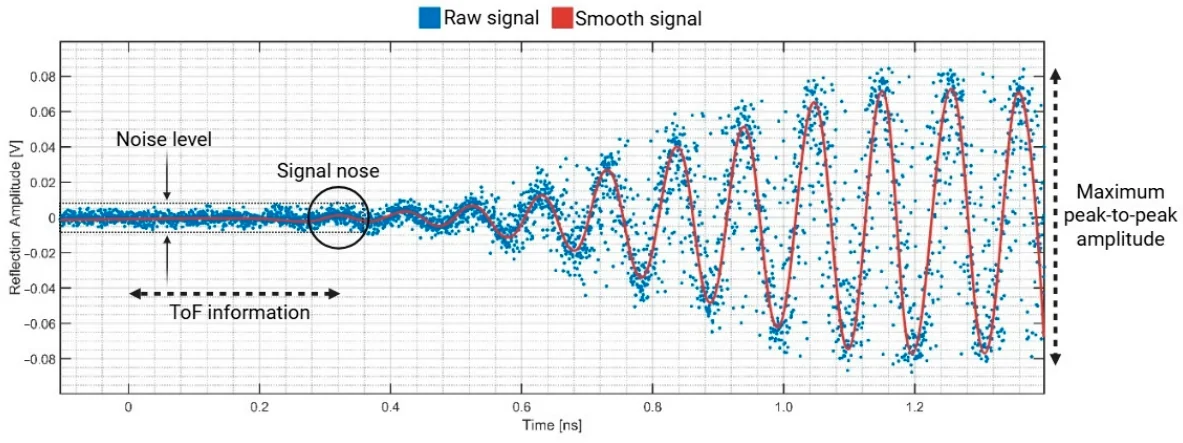
Raw and smoothed reflection signal. Signal features such as time-of-flight (ToF) and maximum reflection amplitude are shown as well.

The raw reflection signal was smoothed using a histogram-based technique. As shown in Figure 6, the initial part of the signal exhibits fluctuations around zero. Although the reflection amplitude in this region is small, the noise level can lead to incorrect identification of the signal nose and, consequently, inaccurate ToF estimation. To address this, a reflection amplitude threshold of 10 mV was defined to ensure reliable detection of the first distinct peak of the sinusoidal signal. Subsequent signal processing steps, including feature extraction, were performed following the same procedures described in the previous section.

After extracting the signal features, a low-pass Butterworth filter with a cutoff frequency of 0.5 Hz was applied to remove high-frequency components arising from respiration and motion artifacts. The most suitable signal feature was then selected for regression analysis to evaluate how the reflection signals could be calibrated for absolute IAP estimation rather than relative IAP monitoring. The ten experimental repetitions were divided into two groups: a learning set (five repetitions) and a testing set (five repetitions). First- and second-order regression models were fitted using the learning dataset. The resulting regression coefficients were subsequently applied to the testing dataset to derive absolute IAP values from the corresponding signal features.

Following the absolute IAP estimation from the reflection signals, further statistical analyses were conducted in accordance with the research guidelines of WSACS [1]. In this context, Lin’s correlation analysis was used to assess the absolute agreement between the reference IAP (IAP_Ventra_) and the estimated IAP via the radar sensor (IAP_TRM_). The bias is defined as the mean difference between IAP_Ventra_ measurement and the value obtained from IAP_radar_. The precision was quantified as the standard deviation of this bias, and the limits of agreement were determined as the bias ± 1.96 times the precision, following the Bland–Altman methodology [34]. The percentage error was calculated as twice the precision divided by the mean IAP value measured by each technology. To assess each method’s ability to track temporal changes in IAP (ΔIAP), a four-quadrant concordance analysis was performed. For this purpose, ΔIAP_radar_ was plotted against the corresponding ΔIAP_Ventra_ over the same interval. The concordance rate was defined as the proportion of pairs exhibiting the same direction of change, after excluding pairs in which the absolute changes in both IAP_radar_ and IAP_Ventra_ were ≤2.5 mmHg (or <15%), and pairs in which either ΔIAP_radar_ or ΔIAP_Ventra_ was equal to zero [35]. Additionally, error grid analysis was conducted for the radar sensor to evaluate the potential clinical risk associated with inaccurate measurements, leading to wrong treatment decisions. Statistical analyses were performed with MATLAB (The MathWorks Inc., Natick, MA, USA).

### 2.3. Clinical Perspective of the Modelling and Experimental Design

The simulated IAP levels of 10, 15, 20, and 25 mmHg were selected to span baseline IAP through IAH grades I–III, while the corresponding changes in abdominal geometry were based on clinical observations of how the abdominal wall responds to increasing IAP. The phantom experiments covered a broader IAP range of 5–35 mmHg, extending from normal pressure to severe IAH and ACS-relevant pressures, while reproducing the expected reduction in abdominal compliance with increasing pressure. The phantom represented a patient in the supine position, and the radar sensor was directed toward the umbilical region. Respiratory variation was simulated at 12 breaths/min by periodic pressure modulation of the phantom, whereas cardiac motion was not included. Radar signals and reference IAP were recorded continuously, and low-pass filtering was applied to suppress faster fluctuations and focus the analysis on slower IAP trends. The investigated 5–20 GHz frequency range was selected to evaluate the practical trade-off between tissue penetration, sensing area, wavelength, and electromagnetic energy deposition for localized contact-free monitoring over the umbilical region. Lower frequencies provide greater penetration but larger wavelengths and sensing areas, whereas higher frequencies provide more localized sensing at the cost of reduced penetration into deeper abdominal wall layers. For clinical interpretation, Lin’s concordance correlation coefficient was used to assess agreement between radar-estimated and reference IAP values, while bias represented systematic over- or underestimation and precision reflected the variability of the measurement error. The limits of agreement indicate the expected range of differences for individual measurements. Percentage error provides an overall measure of relative measurement variability, whereas the four-quadrant concordance analysis evaluates the ability of the system to track the direction of IAP changes. Error-grid analysis was used to assess the potential clinical consequence of measurement errors with respect to IAP-related decision thresholds.

## 3. Results

### 3.1. In Silico Results

**Figure 7:**
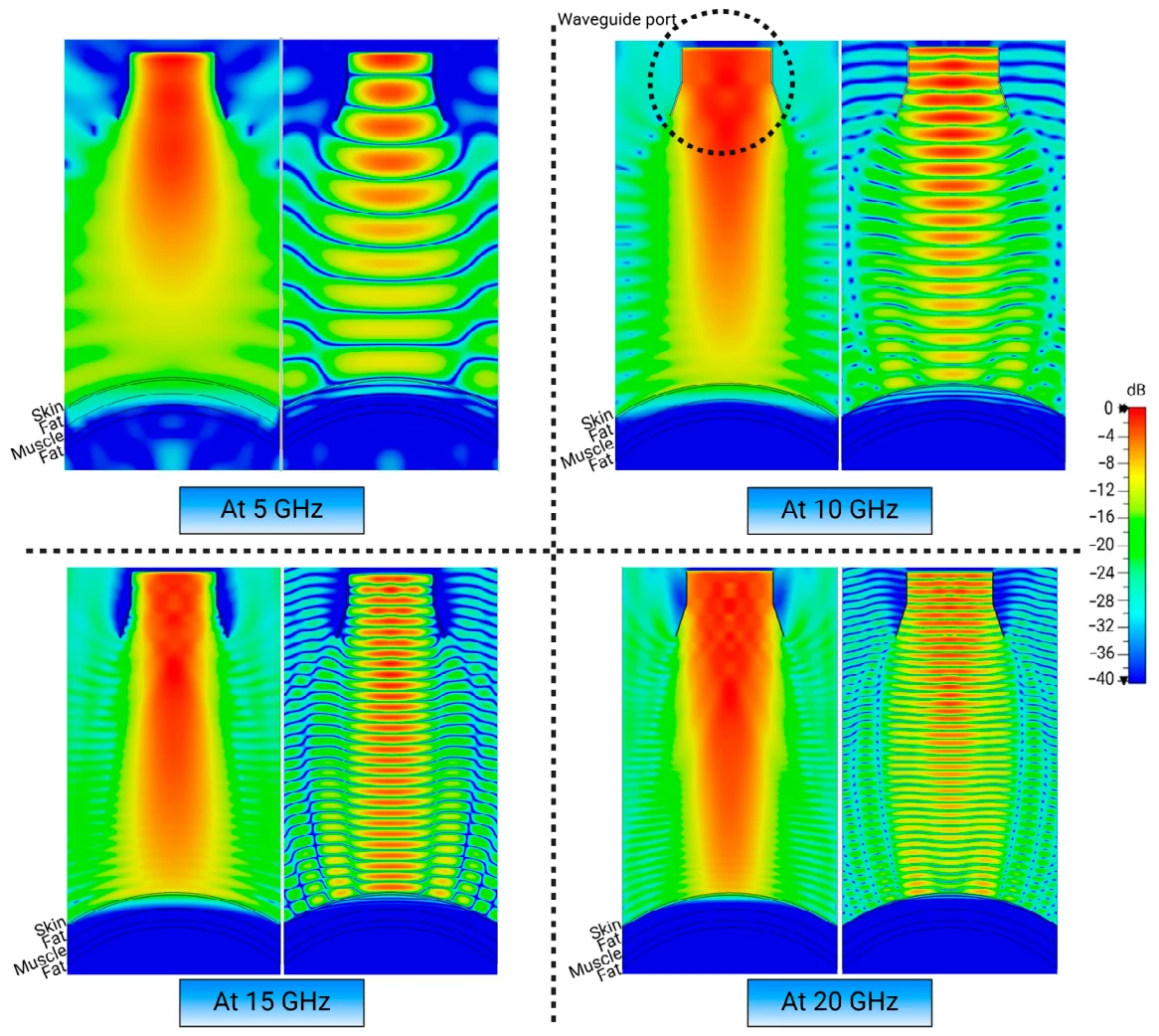
Normalized electric-field and power flow in the multilayer abdominal model at 5, 10, 15, and 20 GHz.

The normalized electric field and power distribution across the abdominal wall layers at baseline IAP are shown in Figure 7 for the four considered frequencies: 5, 10, 15, and 20 GHz. At 5 GHz, the power penetrating into the skin is approximately −16 dB relative to the incident power, followed by −24 dB in the subcutaneous fat and −30 dB in the muscle layer. At 10 GHz, a similar pattern is observed, although attenuation in the skin and fat layers is more pronounced. While the penetrated power in the skin and fat remains around −16 dB and −25 dB, respectively, power reaching the muscle layer becomes negligible (−36 to −40 dB). At higher frequencies, attenuation in the skin increases further, resulting in penetration levels of approximately −32 dB and −34 dB in the fat layer at 15 and 20 GHz, respectively.

**Figure 8:**
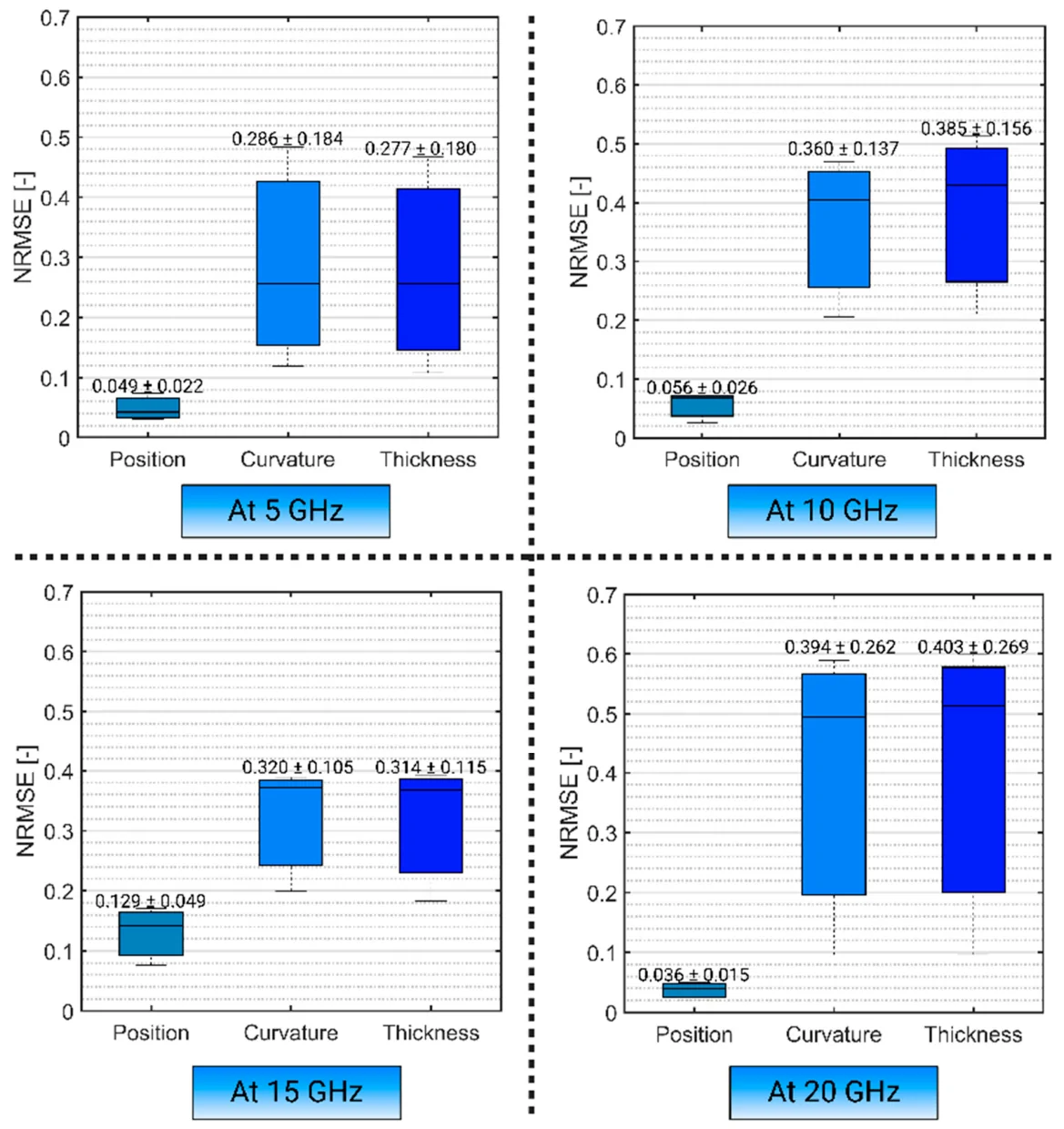
Examination of the abdominal influencing factors on its reflection response. Using the normalized root-mean-square error, the similarity between univariate and multivariate signals was evaluated.

Next, the influence of each morphological parameter on the reflection response of the abdominal wall was evaluated. Figure 8 presents the mean and standard deviation of the normalized root mean squared error at 10, 15, 20, and 25 mmHg, comparing overall reflection changes when thickness, curvature, and distance were varied simultaneously with the cases in which only a single parameter was altered (global vs. local sensitivity). Results are shown for all investigated frequencies.

It is evident that abdominal wall displacement (sagittal abdominal diameter) is the most influential parameter affecting the transient reflection signal. The effects of thickness and curvature did not differ significantly (*p* = 0.85).

**Figure 9:**
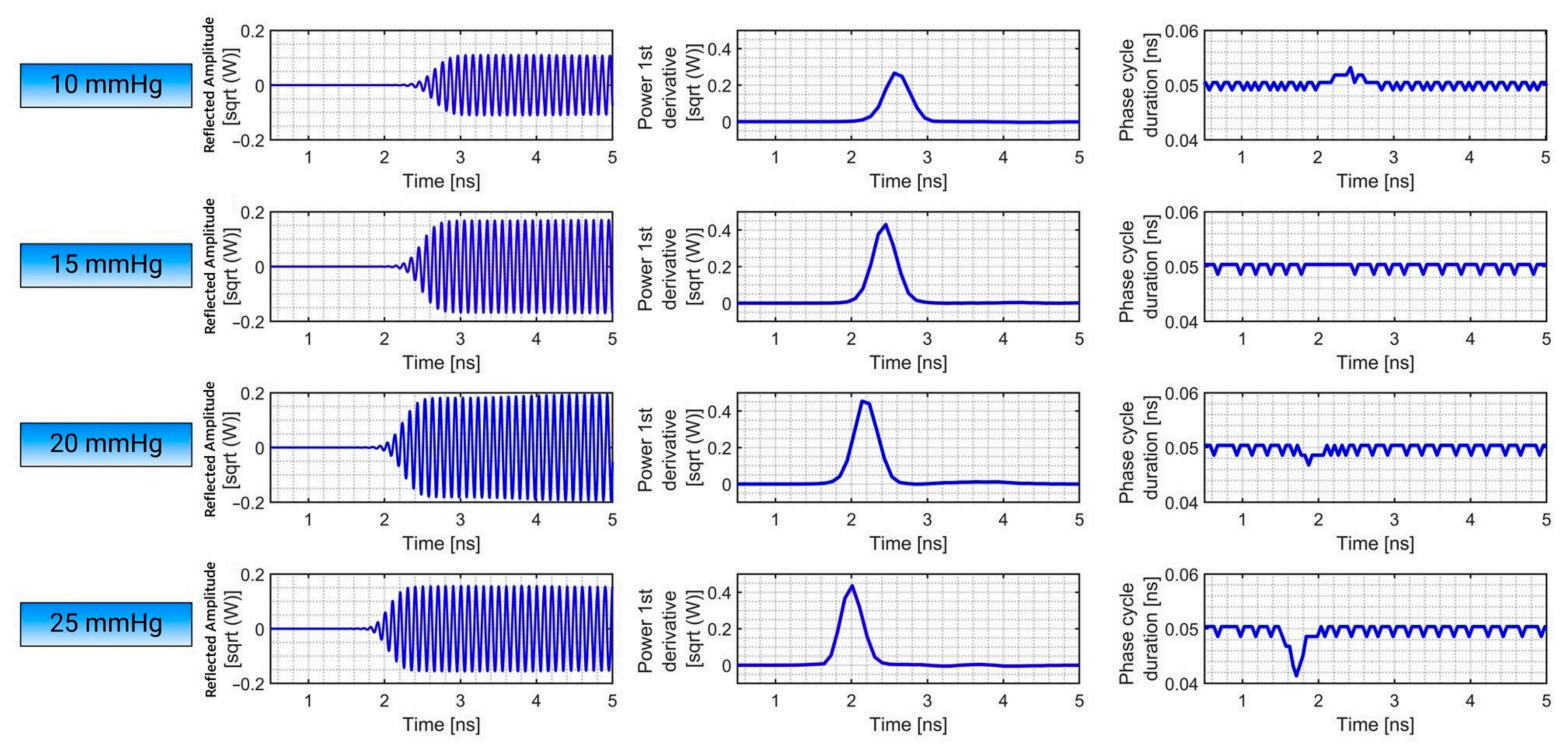
Evolution of reflection response features versus IAP at 10 GHz. The reflection amplitude showed periodic behavior with IAP. The signal phase showed a perturbation when microwaves hit the skin; therefore, it is redundant once the time-of-flight (ToF) is extracted.

Next, the reflection signal features, including maximum peak-to-peak reflection amplitude, ToF, and signal phase, were analyzed across the abdominal wall models representing different IAP levels (see Figure 9).

In Figure 9, the first column shows how the overall reflection amplitude varies with IAP. As observed, the reflection amplitude increases from 10 to 20 mmHg, but decreases when the IAP rises from 20 to 25 mmHg. This periodic behaviour of the reflection amplitude is related to the abdominal wall displacements, which are multiples of the half wavelength values of the used EM waves. For 10 GHz waves propagating in free space the half wavelength equals 1.5 cm. Hence every 1.5 cm displacement of the abdominal wall leads to a quasi-periodic signal due to the round trip pathway. When the abdominal wall displaces towards the EM transmitter and receiver, the reflection amplitude will increase due to decreased beam divergence. The second column presents the first derivative of the reflection response. The peak in the first derivative corresponds to the midpoint of the signal rise time and represents the ToF, indicating the moment when the microwaves reach the abdominal wall model and reflect back. As IAP increases, ToF decreases, which is consistent with the abdominal wall being displaced closer to the transmitting antenna. The third column shows the reflection signal phase at the receiver port. A distinct phase disturbance can be observed at the moment corresponding to the signal nose. Although this feature conveys similar information to the ToF, it was examined to assess whether layer-specific reflections from within the abdominal wall could be identified. However, due to the strong reflection at the air–skin interface, additional sub-reflections from deeper layers are not apparent.

**Figure 10:**
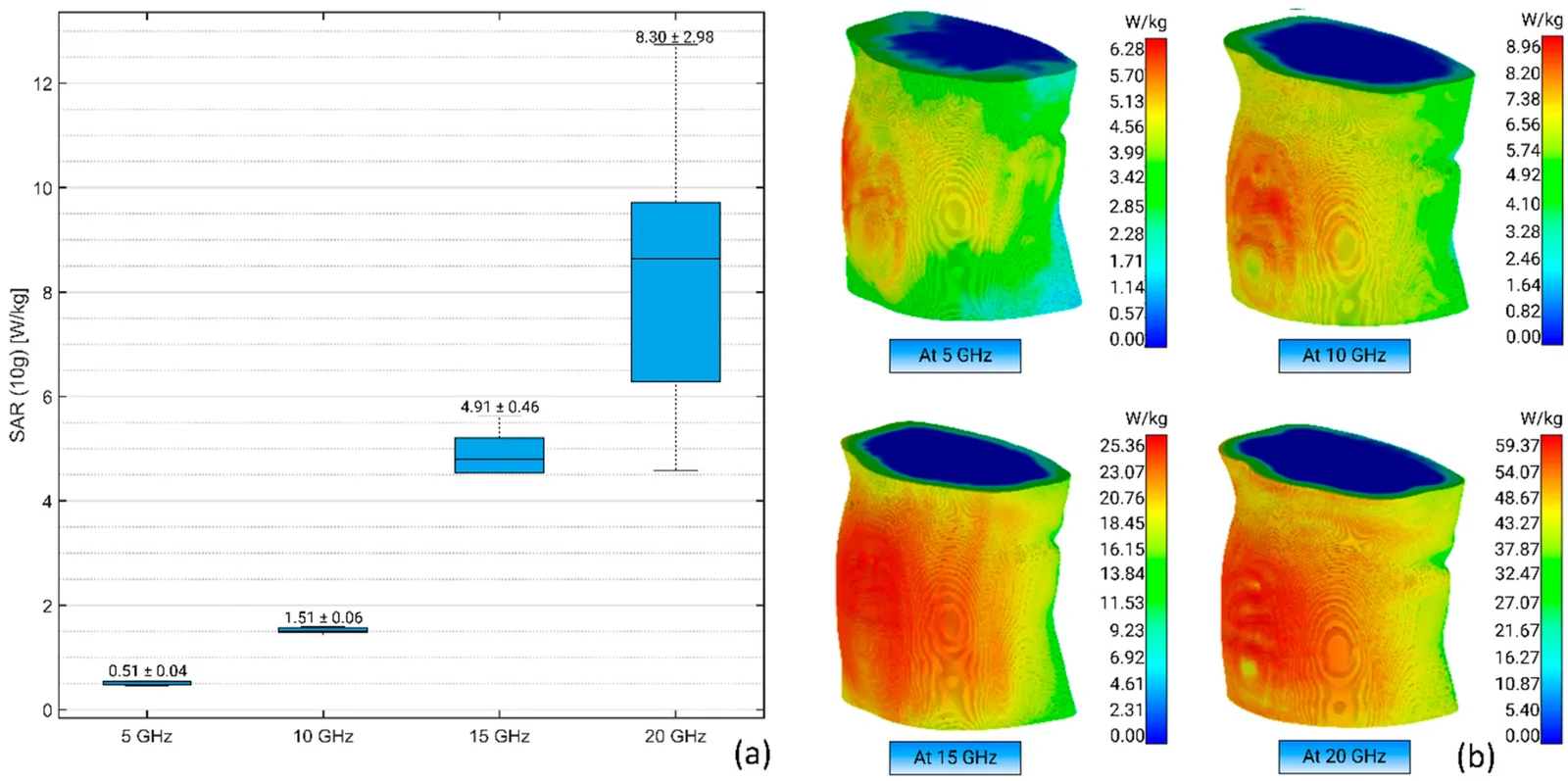
Specific absorption rate (SAR) visualization in virtual subjects studied in this study. (**a**) By increasing the radiation frequency, power deposition happens at lower tissue depths; therefore, a relatively higher SAR is obtained by increasing the frequency. (**b**) Visualization of the deposited energy in the umbilical region.

Additional simulations were performed to evaluate energy deposition in different tissues and to assess SAR for safety considerations. Figure 10 presents the box plot of SAR values averaged over five virtual human subjects.

The deposited energy, and consequently the SAR, increased as the radiation frequency rose from 5 to 20 GHz. In addition, greater within-subject variability in SAR was observed at the higher frequencies.

### 3.2. In Vitro Results

**Figure 11:**
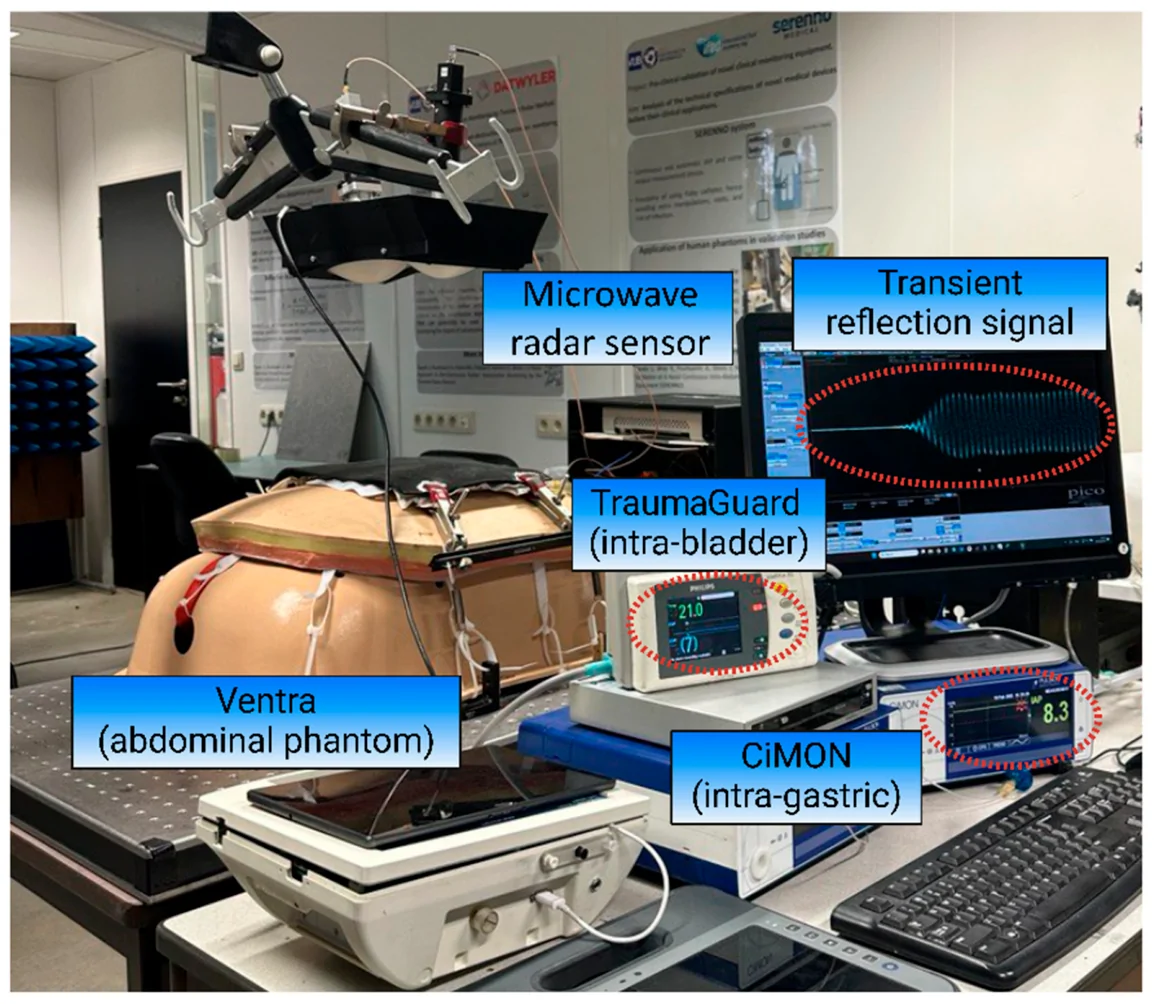
Radar sensor testing on a bench-top abdominal phantom.

The second part of the study involved testing a microwave radar sensor on a bench-top abdominal phantom (Ventra) to monitor IAP using the reflection response. The experimental setup is shown in Figure 11.

Based on the simulation findings from the previous section, the phase information was found to be redundant, as it conveyed essentially the same information as the ToF. Therefore, in the in vitro experiments, only the reflection amplitude and ToF were examined as IAP varied.

**Figure 12:**
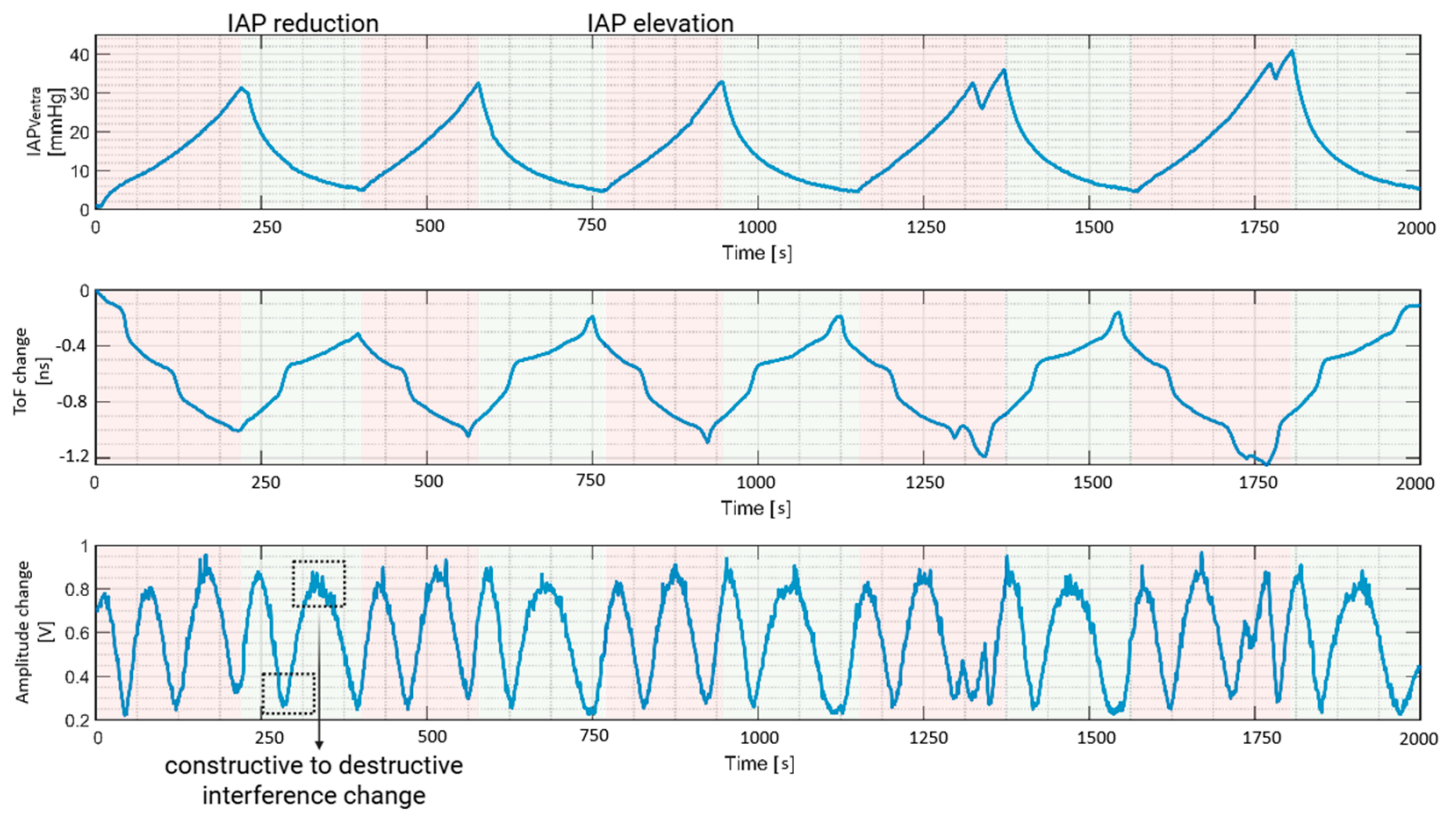
Sampled IAP by Ventra in addition to the time-of-flight (ToF) information and reflection amplitude.

The sampled IAP in Ventra (IAP_Ventra_), along with the corresponding reflection amplitude and ToF changes, is shown in Figure 12.

Overall, fluctuations in IAP are accompanied by opposite changes in the position of the reflection nose, which are caused by changes in the sagittal abdominal diameter. The reflection amplitude, however, exhibits a periodic pattern, increasing and decreasing even when IAP is changed monotonically. This behavior is consistent with the simulation results presented and discussed in the previous section, where a periodic trend in reflection amplitude was observed as IAP increased from 10 to 25 mmHg. We observe that the peak amplitude values increase each period when the abdominal wall moves towards the antennas. Based on these observations, the ToF feature was selected as the primary parameter for regression analysis and absolute IAP estimation.

**Figure 13:**
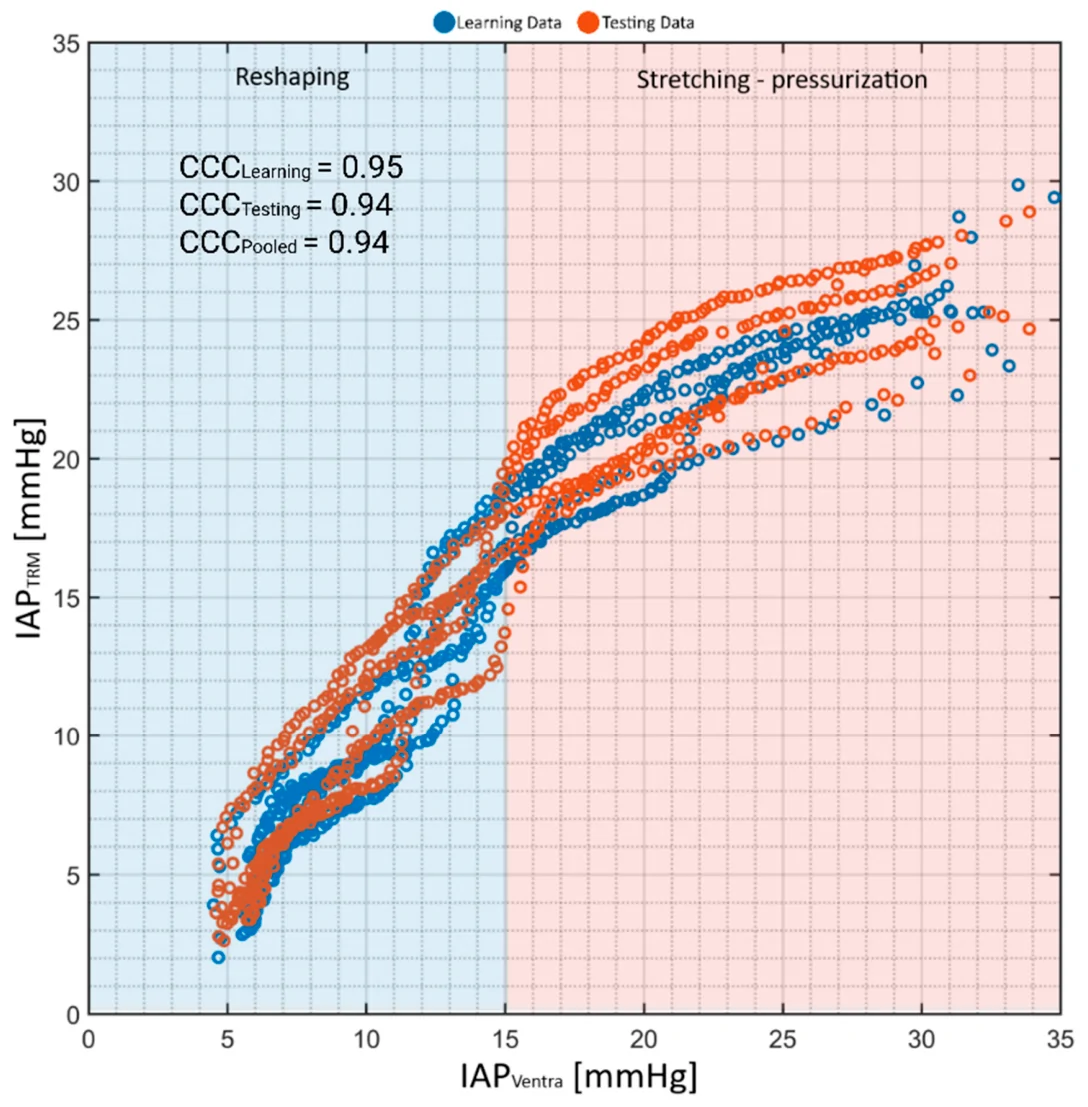
IAP_TRM_ versus IAP_Ventra_ for the learning, testing, and pooled datasets. Lin’s correlation analysis showed an absolute agreement correlation of 0.95, 0.94, and 0.94 for the learning, testing, and pooled datasets, respectively.

Although second-order polynomial fits yielded higher individual R^2^ values (0.97 on average) compared to first-order fits (R^2^ between 0.87 and 0.92), the second-order models exhibited greater variability across datasets. Consequently, when averaged or median coefficients were applied to unseen testing data, the first-order model consistently outperformed the second-order model. Therefore, despite slightly lower individual R^2^ values, the linear model was selected for its robustness and consistency across multiple datasets. The median regression coefficients from the learning dataset were subsequently used to convert the ToF of the testing dataset into absolute IAP values. Figure 13 shows the IAP_TRM_ versus IAP_Ventra_ together with Lin’s correlation coefficient (CCC) for each group of data.

**Figure 14:**
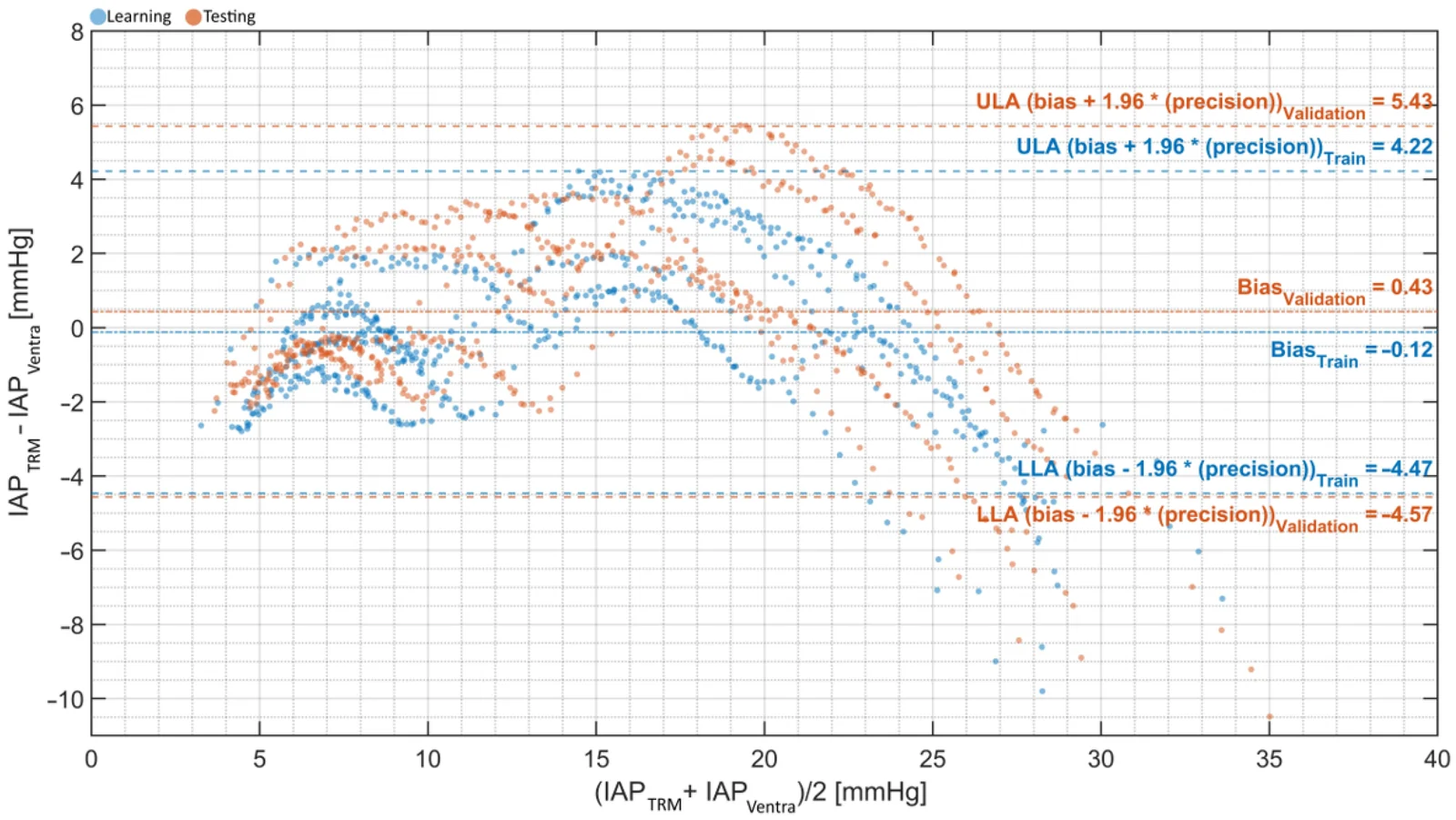
Bland–Altman analysis done on IAP_TRM_ with respect to IAP_Ventra_. Bias, as well as the upper and lower limits of agreement (ULA and LLA), are indicated.

After estimating the absolute IAP, Bland–Altman analysis (Figure 14) was performed to assess bias, precision, limits of agreement, and percentage error.

The numerical results of the Bland–Altman analysis are depicted in Table 3.

**Figure 15:**
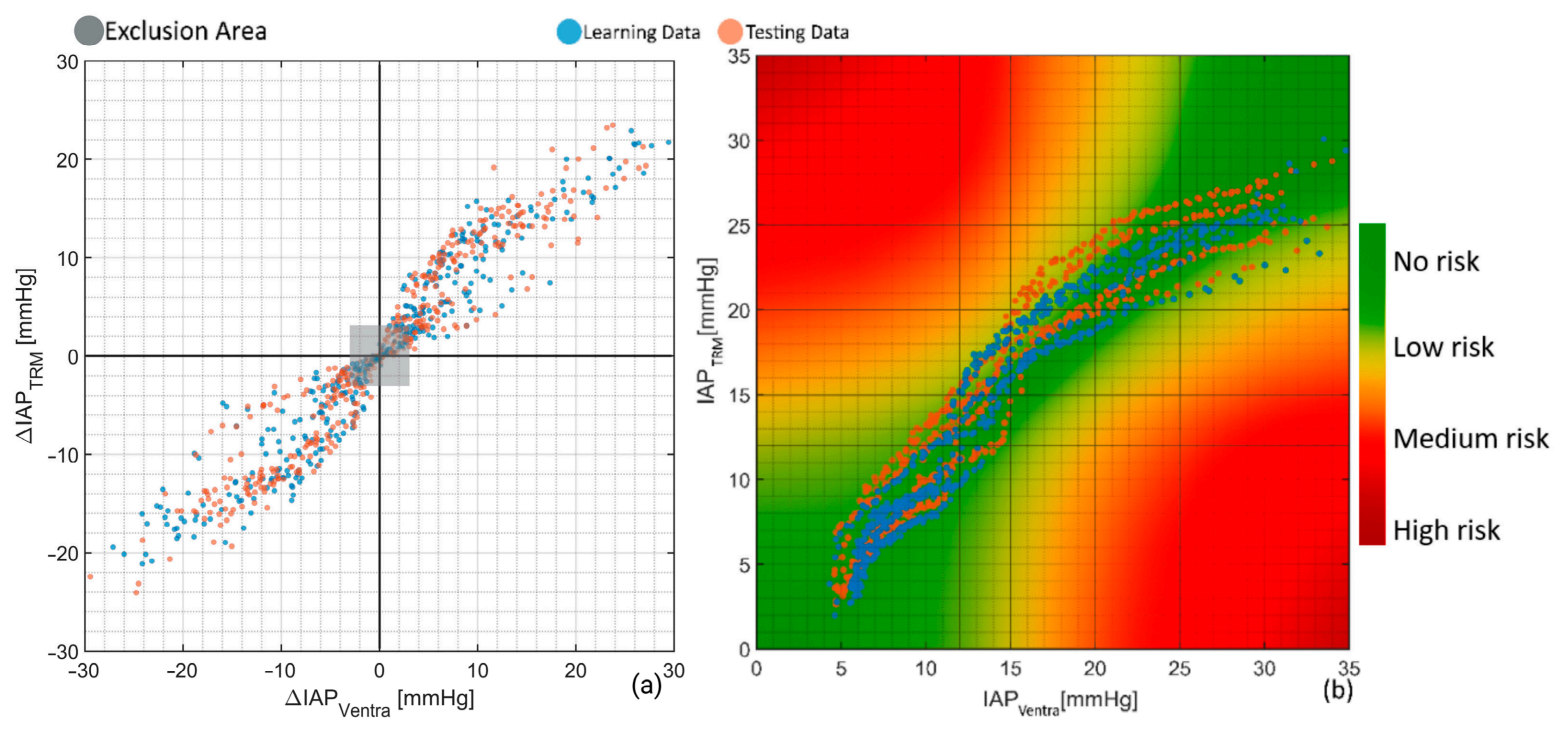
Results of concordance and error-grid analyses. (**a**) After exclusion of 19.1% of paired changes according to the predefined exclusion criteria, all retained changes showed directional agreement, corresponding to a concordance rate of 100% (95% CI: 99.4–100%). (**b**) Error-grid analysis revealed that 71.3% of measurements were in the no-risk and 28.7% in the low-risk region.

The robustness of tracking IAP changes was evaluated using concordance analysis (Figure 15). After excluding 19.1% of paired changes according to the predefined exclusion criteria, all retained changes showed agreement in direction, corresponding to a concordance rate of 100% (95% CI: 99.4–100%). This indicates strong reliability in detecting directional changes in IAP. Lastly, an error-grid analysis was performed to evaluate the potential clinical risk associated with incorrect treatment decisions arising from inaccurate IAP measurements. In this regard, 71.3% results were in the no-risk region, while 28.7% of the results were in the low-risk region (Figure 15).

## 4. Summary and Discussion

### 4.1. Overall Summary

This study provided a first fundamental investigation of how morphological changes in the abdominal compartment due to IAH influence its microwave reflection response, and identified the key contributing factors through multivariate and univariate sensitivity comparisons. Reflection signal features, including maximum peak-to-peak amplitude, ToF derived from the signal nose, and signal phase state, were evaluated across different simulated IAP levels to determine the most suitable feature for extracting absolute IAP values. In addition, SAR was simulated and calculated in five virtual subjects with varying BMIs. Building on these in silico findings, a transient microwave radar sensor was developed and tested on a bench-top abdominal phantom to monitor IAP. Guided by insights from the simulation phase, reflection amplitude and ToF were examined as functions of IAP. Ten experiments were conducted, with 50% used to perform regression analysis and learn how to translate changes in reflection signal features to absolute IAP, and the remaining 50% used to test the calibration approach within the testing dataset.

Numerical simulations demonstrated that at 5 and 10 GHz, incident power undergoes substantial attenuation as it propagates through tissue, and that at 10 GHz, the increased attenuation in superficial layers results in negligible penetration into muscle tissue. At higher frequencies (15–20 GHz), attenuation becomes even more pronounced, further reducing deeper tissue penetration. Sensitivity analyses indicated that abdominal wall displacement (sagittal abdominal diameter) has the dominant influence on the reflected signal, largely overshadowing the effects of curvature and thickness changes. Among the evaluated signal features, phase information was found to convey essentially the same information as ToF, rendering it redundant when ToF is already available. Reflection amplitude exhibited a periodic increase–decrease pattern with varying IAP, challenging its use as a single reliable feature, particularly over large pressure ranges. In combination with ToF it can yield new insights in curvature and thickness changes. The numerically simulated SAR values remained below the applicable ICNIRP exposure limits under the investigated conditions.

The in vitro testing and subsequent calibration demonstrated that the radar sensor estimated IAP with a bias of 0.43 mmHg and a precision of 2.55 mmHg. The limits of agreement were −4.57 to 5.43 mmHg, with a percentage error of 33.49%. All retained changes after application of the predefined exclusion criteria showed agreement in the direction of IAP change. The majority of the results (71.3%) were in the no-risk region, while the remaining 28.7% were in the low-risk zone.

### 4.2. Discussion

#### 4.2.1. Technical Interpretation and Sensor Performance

This study’s output provided fundamental information on how the radiation frequency alters penetration depth and spot size (lateral resolution), therefore being helpful for selecting a frequency band for future microwave sensor development for diagnosis purposes, including IAP measurement and IAH detection. In fact, the most suitable frequency band (for narrowband radiation) is a trade-off among the required penetration depth, spot size, and hardware costs and dimensions. For such a purpose the spot size should preferably be in the order of magnitude of the abdominal wall dimensions. Taking the hardware size and cost, as well as the antenna aperture into account, 8–12 GHz seems to be a good choice. Nevertheless, if more penetration is needed to extract more in-depth information, the carrier frequency should be lowered. Conversely, extending the operating frequency above 20 GHz would reduce the wavelength and sensing area, potentially improving spatial localization and enabling more compact antenna hardware. However, the reduced penetration depth would make the response increasingly dominated by superficial tissue interfaces and small surface displacements, limiting the extraction of deeper abdominal-wall information. In addition, the increasingly superficial electromagnetic energy deposition at higher frequencies requires careful consideration of local tissue exposure and applicable safety limits. Therefore, frequencies above 20 GHz were not prioritized in this exploratory study, which focused on balancing penetration depth, spatial resolution, and electromagnetic exposure for abdominal-wall sensing. Regarding the influencing factors, we observed that the abdominal wall displacement will have the most impact on defining the reflection response of the abdominal wall (five to six times greater than curvature and thickness impact). Therefore, in cases where time-domain microwave reflectometry sensors are designed to monitor the abdominal compartment and extract IAP-related information, the signal feature to be focused on would be the distance change. Abdominal wall thickness changes, therefore, are recommended to be tracked by using a distance compensated contact-free measurements or by using a contact-based microwave sensor; excluding any displacement impact to mask the thickness changes information. Regarding the curvature or abdominal shape monitoring, a similar strategy should be used to have displacement-compensated amplitude measurements or exclude the impact of displacement. In such a case, the emitter and receiver antennas should be on a movable linear stage that is synchronized with the abdominal wall movement. Thus, the distance between the umbilical region and the antennas will be always fixed. An important limitation of ToF-based monitoring is its sensitivity to global patient movement, since changes in body position or bed inclination can alter the antenna-to-abdominal-wall distance and mimic IAP-related displacement. Rapid ToF changes, occurring on a shorter timescale than typical IAP variations, could be identified as motion artifacts and rejected using appropriate temporal filtering. However, slower patient shifts require an independent motion reference. One approach would be a differential radar configuration with a second channel directed toward a body region minimally affected by IAP, allowing common patient motion to be identified and compensated. An optical reference could additionally help ensure consistent positioning of the measurement spot.

Generally, if the sensing objective is limited to tracking sagittal abdominal displacement, other contact-free technologies such as optical depth sensing, laser-based displacement measurement, or digital image correlation could in principle provide similar surface-motion information. The present study was intended as an exploratory investigation of the mechanisms underlying microwave-based IAP sensing rather than as a comparison between displacement-sensing technologies. One potential advantage of microwave reflectometry is its ability to provide subsurface dielectric and geometric information in addition to surface-distance tracking. In the present setup, however, the strong reflection at the air–skin interface limited the extraction of deeper-layer information. Future systems using lower frequency ranges and/or displacement compensation may allow simultaneous assessment of abdominal-wall motion and deeper tissue-related features.

Regarding the SAR values, it should be emphasized that the reported simulations were normalized to 1 W of continuous-wave radiation. In contrast, the developed laboratory prototype operated at a peak transmitter output power of 0.3 W with a 50% duty cycle. Therefore, the actual time-averaged exposure associated with the prototype is expected to be substantially lower than the simulated 1 W continuous-wave condition. Nevertheless, this safety assessment is based on numerical simulations and power-scaling considerations was not verified by direct experimental SAR measurements. Moreover, the present simulations did not specifically model more specific cases such as pregnant anatomy or the presence of implanted medical devices. These conditions may alter the exposure scenario or introduce additional safety considerations and therefore require dedicated assessment before clinical use.

In vitro experiments showed similar trends for the maximum peak-to-peak reflection amplitude and ToF, including the periodic amplitude variation associated with abdominal-wall displacements corresponding to approximately half-wavelength intervals. These findings further support the use of ToF as the primary feature for tracking abdominal-wall displacement. To further improve the system’s performance, adaptive filtering, particularly when combined with an independent respiratory or motion-reference signal, may suppress artifact-related ToF variations and measurement noise. Multi-frequency illumination may also provide complementary information with different penetration depths and wavelength-dependent sensitivities, potentially improving measurement robustness and precision of the current study.

#### 4.2.2. Clinical Implications

Examination of the Bland–Altman results and comparison with the thresholds defined by WSACS showed that the bias was within the acceptable limit (<1 mmHg), whereas the precision and limits of agreement slightly exceeded the recommended margins of 2 and 4 mmHg, respectively. Clinically, the testing bias of 0.43 mmHg indicates minimal systematic error, while the precision of 2.55 mmHg means that individual measurements may still differ from the reference by several mmHg. Therefore, the current performance is more suitable for trend monitoring and recognition of broader transitions toward IAH, whereas measurements close to clinically important thresholds should be interpreted cautiously and confirmed by intravesical measurement before high-stakes treatment decisions are made. At its current stage, the system is therefore best suited as an adjunct monitoring and decision-support tool for early detection of rising IAP, monitoring response to therapy, and identifying patients at increasing risk of IAH or ACS, rather than as a standalone replacement for intravesical IAP measurement.

Overall, substantial clinical investigation is still required to characterize the reflection response of the abdominal compartment across a large and diverse patient population with varying body anthropometrics, using paired radar and intravesical IAP measurements to determine a more robust calibration strategy. When combined with learning-based models, these systems could support the development of a robust non-invasive alternative to current invasive IAP measurement techniques. Realistic ICU implementation would also require stable sensor positioning, appropriate motion compensation, and a standardized calibration strategy. Moreover, practical parameters such as the required recalibration frequency, suitable abdominal sensing locations, sensitivity to sensor repositioning, and the definition of a standardized clinical measurement protocol still need to be established before clinical implementation.

### 4.3. Limitations

The first limitation concerns the abdominal wall models used in the numerical simulations. Although the CAD models were based on available anatomical, mechanical, and electromagnetic data, several simplifications were made. One key simplification was assuming that each tissue layer was homogeneous, while real human tissues show clear variations within a layer. Moreover, the layer composition may also vary over time as patients may accumulate fluids in the different layers due to the inflammatory response. Another simplification was assuming isotropic properties, even though some tissues, especially muscles, exhibit direction-dependent mechanical and electromagnetic behavior. Although tissue anisotropy and fluid accumulation may alter the local dielectric permittivity and conductivity of the abdominal wall, their direct effect on the ToF feature used in this study is expected to be limited because ToF was derived from the first reflection at the air–skin interface and therefore predominantly represents the antenna-to-skin propagation distance. Nevertheless, such changes may indirectly affect ToF through alterations in abdominal-wall geometry and may influence the reflected waveform and amplitude. Their impact would become more important when analyzing signals propagating within deeper tissue layers. Future studies could include anisotropy, which may also provide useful information for IAP monitoring, as changes in the sagittal and coronal planes may differ. Mechanical stretching of the abdominal wall was also not included in the models. Adding this factor in future simulations would make the models more realistic. Regarding the frequency selection, this study used four frequencies based on expected penetration depth and spot size. Future work could include a wider range, including lower frequencies (such as around 500 MHz), to explore whether layer-specific information can be extracted. This may also have value for body composition studies, such as estimating fat and muscle thickness. For the SAR analysis, the main limitation was the small number of virtual subjects and limited variation in the BMI values. The prototype operated under substantially lower time-averaged exposure than the simulated 1 W continuous-wave condition; nevertheless, including more subjects would provide a more reliable characterization of SAR variability. The SAR simulations were performed using the antenna-to-skin distance corresponding to an IAP of 25 mmHg, representing the maximum abdominal distension considered in this study. However, the reported SAR values may still depend on antenna-to-skin distance. Distance does not directly enter the SAR calculation, but it can alter the spatial distribution and magnitude of the electric field within the tissues. Future studies should therefore evaluate SAR across different antenna-to-skin distances. In addition, the SAR assessment was based on numerical simulations and was not validated by direct experimental dosimetric measurements; therefore, the safety interpretation is limited to the simulated exposure conditions. For the in vitro tests, the main limitation is that all experiments were performed on a single artificial subject with static layer composition. As a result, natural variations in body shape, tissue thickness, and anatomy could not be considered. Other patient-specific variations, such as abdominal wall edema and postoperative changes in the abdominal compartment, were not represented in the current study. This also means that the calibration results are likely more accurate for this specific phantom than they would be for a broader population. In addition, the number of repeated phantom experiments was limited, with only five repetitions used for model learning and five for testing. Therefore, the regression and validation results should be considered preliminary, and a larger number of independent experiments will be required to more reliably assess model performance and generalizability. Similar to the in silico models, the abdominal phantom used here had isotropic and homogeneous layers, which is another limitation since real tissues show more variation. Another aspect to be considered in future studies is that some patients may have medical equipment, such as drains, within the measurement area on the abdominal wall, which may prevent measurements from being performed at the umbilical region. In this regard, the feasibility of placing the electromagnetic probes at alternative abdominal locations, together with the corresponding signal-processing requirements, should also be investigated. Similarly, the presence of patient dressings should be taken into account. Dressings may introduce additional attenuation and could affect reliable ToF extraction. They may also alter the microwave reflection amplitude and therefore represent another potential source of measurement variability. Although standardized IAP measurements are performed in the absence of abdominal muscle contraction, spontaneous abdominal muscle activity may still occur in clinical practice and could influence the radar response. Its impact was not specifically evaluated in the present phantom study and should therefore be investigated in future clinical validation. Therefore, it should be noted that the abdominal phantom used in this study did not fully reproduce the complexity of real clinical conditions. Another limitation is that the combined use of reflection amplitude and ToF information was not fully exploited in the present analysis. In principle, ToF mainly provides information on changes in propagation distance, whereas reflection amplitude is affected not only by displacement-related beam divergence but also by changes in abdominal wall shape and layer thickness. By normalizing displacement-related amplitude changes and correcting them for shape-induced variations in reflection amplitude, it may be possible to separate pure distance effects from abdominal wall thickness and curvature effects. This combined interpretation could therefore provide additional information on IAP-related abdominal wall thickness changes and shape changes. Future studies should investigate this combined amplitude-ToF approach, as it may improve the physiological interpretation of the sensor response and enhance the robustness of IAP monitoring across different anatomies.

## 5. Conclusions

This study highlights key aspects of using contact-free microwave reflectometry for non-invasive indirect IAP monitoring and can serve as a reference for future work. Sagittal abdominal diameter was identified as the dominant morphological factor influencing the reflection response. Phantom testing supported ToF-based IAP tracking. In clinical practice, the technology is currently more appropriate for early warning and monitoring IAP trends than for obtaining precise absolute IAP values, and it is not yet a substitute for standard intravesical measurement. Its ability to support clinical decision-making, including guiding fluid therapy, requires prospective validation in patients.

## Figures and Tables

**Table 1:** Thickness and mechanical properties of each abdominal wall layer used in the baseline CAD model.

Tissue	Thickness [cm]	Density [g/cm^3^]	Young’s Modulus [kPa]	Poisson’s Ratio [−]
Skin	0.2	1.0	2100.0	0.5
Subcutaneous fat	1.3	0.9	3.3	0.3
Muscle	1.0	1.1	110.0	0.3
Pre-peritoneum fat	0.7	0.9	3.3	0.3
Peritoneum	0.1	1.0	940.0	0.5

**Table 2:** Dielectric permittivity and electric conductivity of each abdominal wall layer at 5, 10, 15, and 20 GHz.

Tissue	Relative Permittivity [−]	Electrical Conductivity [S/m]	Complex Permittivity [−]
5 GHz			
Skin	35.8	3.0	35.8–11.0 j
Fat	10.1	0.7	10.1–2.4 j
Muscle	49.5	4.0	49.5–14.5 j
Preperitoneal fat	10.1	0.7	10.1–2.4 j
Peritoneum	79.6	5.4	79.6–19.3 j
10 GHz			
Skin	31.3	8.0	31.3–14.4 j
Fat	8.8	1.7	8.8–3.1 j
Muscle	42.8	10.6	42.8–19.1 j
Preperitoneal fat	8.8	1.7	8.8–3.1 j
Peritoneum	67.6	18.0	67.6–32.4 j
15 GHz			
Skin	26.4	13.8	26.4–16.5 j
Fat	7.8	2.8	7.8–3.3 j
Muscle	36.4	17.9	36.4–21.5 j
Preperitoneal fat	7.8	2.8	7.8–3.3 j
Peritoneum	54.5	31.9	54.5–38.2 j
20 GHz			
Skin	22.0	19.2	22.0–17.3 j
Fat	7.0	3.7	7.0–3.3 j
Muscle	31.0	24.7	31.0–22.2 j
Preperitoneal fat	7.01	3.7	7.0–3.3 j
Peritoneum	43.4	43.8	43.4–39.4 j

**Table 3:** Bland–Altman results of the estimated IAP with the radar sensor.

Study Group	Mean IAP (mmHg)	Bias (mmHg)	Precision (mmHg)	LLA (mmHg)	ULA (mmHg)	PE (%)
Learning (*n* = 600)	14.32	−0.12	2.21	−4.47	4.22	30.87
Testing (*n* = 600)	15.23	0.43	2.55	−4.57	5.43	33.49
Pooled (*n* = 1200)	14.77	0.15	2.40	−4.55	4.86	32.50

LLA: lower limit of agreement, ULA: upper limit of agreement, PE: percentage error.

## Data Availability

The data generated or analyzed during this study are available on request from the correspondence author.

## Abbreviations

The following abbreviations are used in this manuscript:

ACS	Abdominal compartment syndrome
BMI	Body mass index
CAD	Computer-aided design
CAUTI	Catheter-associated urinary tract infection
CCC	Lin’s concordance correlation coefficient
CLK	Clock signal
CT	Computed tomography
EM	Electromagnetic
FMCW	Frequency-modulated continuous-wave
IAH	Intra-abdominal hypertension
IAP	Intra-abdominal pressure
ICNIRP	International Commission on Non-Ionizing Radiation Protection
ICU	Intensive care unit
IEC	International Electrotechnical Commission
IEEE	Institute of Electrical and Electronics Engineers
LLA	Lower limit of agreement
nRMSE	Normalized root-mean-square error
PE	Percentage error
SAR	Specific absorption rate
SD	Standard deviation
SPST	Single-pole-single-throw
ToF	Time of flight
TRM	Transient radar method
ULA	Upper limit of agreement
VCO	Voltage-controlled oscillator
WSACS	Abdominal Compartment Society (formerly World Society of the Abdominal Compartment Syndrome)
